# Nuclear RNA-seq of single neurons reveals molecular signatures of activation

**DOI:** 10.1038/ncomms11022

**Published:** 2016-04-19

**Authors:** Benjamin Lacar, Sara B. Linker, Baptiste N. Jaeger, Suguna Krishnaswami, Jerika Barron, Martijn Kelder, Sarah Parylak, Apuã Paquola, Pratap Venepally, Mark Novotny, Carolyn O'Connor, Conor Fitzpatrick, Jennifer Erwin, Jonathan Y. Hsu, David Husband, Michael J. McConnell, Roger Lasken, Fred H. Gage

**Affiliations:** 1 Laboratory of Genetics, The Salk Institute for Biological Studies, 10010 N Torrey Pines Road, La Jolla, California 92037-1002, USA; 2 J. Craig Venter Institute, La Jolla, California 92037, USA; 3 Department of Biochemistry and Molecular Genetics, University of Virginia School of Medicine, Charlottesville, Virginia 22908, USA

## Abstract

Single-cell sequencing methods have emerged as powerful tools for identification of heterogeneous cell types within defined brain regions. Application of single-cell techniques to study the transcriptome of activated neurons can offer insight into molecular dynamics associated with differential neuronal responses to a given experience. Through evaluation of common whole-cell and single-nuclei RNA-sequencing (snRNA-seq) methods, here we show that snRNA-seq faithfully recapitulates transcriptional patterns associated with experience-driven induction of activity, including immediate early genes (IEGs) such as *Fos*, *Arc* and *Egr1*. SnRNA-seq of mouse dentate granule cells reveals large-scale changes in the activated neuronal transcriptome after brief novel environment exposure, including induction of MAPK pathway genes. In addition, we observe a continuum of activation states, revealing a pseudotemporal pattern of activation from gene expression alone. In summary, snRNA-seq of activated neurons enables the examination of gene expression beyond IEGs, allowing for novel insights into neuronal activation patterns *in vivo.*

## Abstract (web summary)

The molecular dynamics associated with neuronal activation patterns *in vivo* are unclear. Lacar *et al*. perform single-nuclei RNA-sequencing of hippocampal neurons from mice exposed to a novel environment, and identify large-scale transcriptome changes in individual neurons associated with the experience.

## Graphical abstract

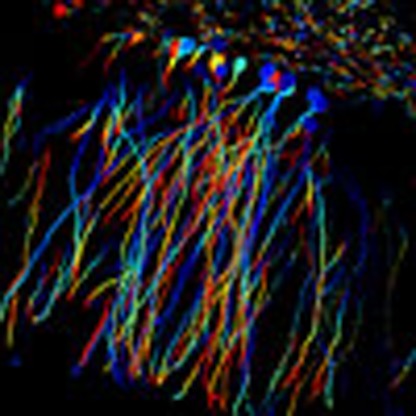

Single-cell RNA-sequencing (RNA-seq) techniques^1^,^2^,^3^,^4^ provide a means to examine the fine-scale differences underlying cell populations that bulk profiling obscures. Due to the high degree of structural and functional heterogeneity that exists between neurons, interest has recently grown to map the landscape of neural cell types^5^,^6^,^7^,^8^,^9^. Single-cell RNA-seq is key for these studies, as it makes possible the sub-classification of cells that otherwise would be indistinguishable based on morphology and anatomical position. For example, the response of individual dentate granule cells (DGCs) to an experience, such as exposure to a novel environment (NE), is highly variable and relatively unpredictable based on current staining approaches^10^. Yet such exposure has a well-documented rapid effect on the neural transcriptome, namely through activation of immediate early genes (IEGs)^11^.

Although understanding the transcriptional response of individual neurons with respect to activity is of compelling interest, it is important to first ensure that the response being measured is evoked by the experience of the animal and not a by-product of the technique. For example, technical procedures that denervate neurons (that is, lesion, transection of dendrites) can elicit IEG expression, mimicking the transcriptional state of an activated neuron^12^. Dendritic loss is also likely to occur during protease dissociation, which is a key component of current single-cell methods. To properly characterize the transcriptome in response to neural activity, these technical issues must be addressed.

Single-nuclei RNA-seq (snRNA-seq) has recently arisen as an alternative to single-cell methods^13^. An advantage of snRNA-seq is a rapid dissociation protocol that does not require either protease digestion or heating, reducing the likelihood of aberrant transcription. Spurious gene expression is also likely to be minimized, since fully mature ribosomes are localized to the cytoplasm^14^. Therefore, even if messenger RNAs (mRNAs) of transcription factors were expressed following dissociation, they would not be translated due to a lack of ribosomes, precluding transcription of their downstream targets. Nuclei preparations also allow for a focused snapshot of the nuclear transcriptome, enabling the identification of nascent, neuronal activity-associated mRNA.

Early activity-induced transcriptional changes have been well characterized within the mammalian brain. Specifically, IEGs including *Fos*^15^,^16^, *Arc*^17^,^18^ and *Egr1* (ref. 19) are rapidly induced after exposure to an activity-inducing experience. Despite this understanding of immediate events, much remains unknown with respect to the heterogeneity of gene expression that is both upstream and downstream of these IEGs.

We characterized the transcriptome from individual activated dentate granule neurons using snRNA-seq. The dentate gyrus (DG), a subregion of the hippocampus, was studied due to its importance in learning and memory^20^,^21^,^22^,^23^ and its unusually sparse activity. Sparsity in the DG is observed both at the level of electrical activity^24^ and through minimal induction of IEG expression^10^. Upon treatment with seizure-inducing drugs such as pentylenetetrazole (PTZ), a majority of DGCs become activated^25^, providing a stark contrast with the normally sparse IEG levels. This ability to investigate DGCs across a dynamic range of activation makes the DG an optimal system to examine the transcriptional response evoked by neural activity.

Our findings demonstrated that IEG expression was consistent with the behavioural experience of the mouse when single nuclei were dissociated from neurons. In addition, we found that large-scale changes in the transcriptional response, revealing a previously unappreciated heterogeneity of activated neurons.

## Results

### PTZ-independent IEG expression in dissociated whole-cell DGCs

To determine whether the common whole-cell dissociation method using papain was suitable for studying activity-induced expression, whole DGCs were dissociated and examined by single-cell RNA-seq. DGCs are marked by PROX1, a transcription factor found almost exclusively in DGCs of the adult brain^26^. The mice used in this experiment expressed cytoplasmic green fluorescent protein linked to the *Prox1* promoter^27^, which enabled sorting for DGCs without permeabilizing the cell.

**Figure 1:**
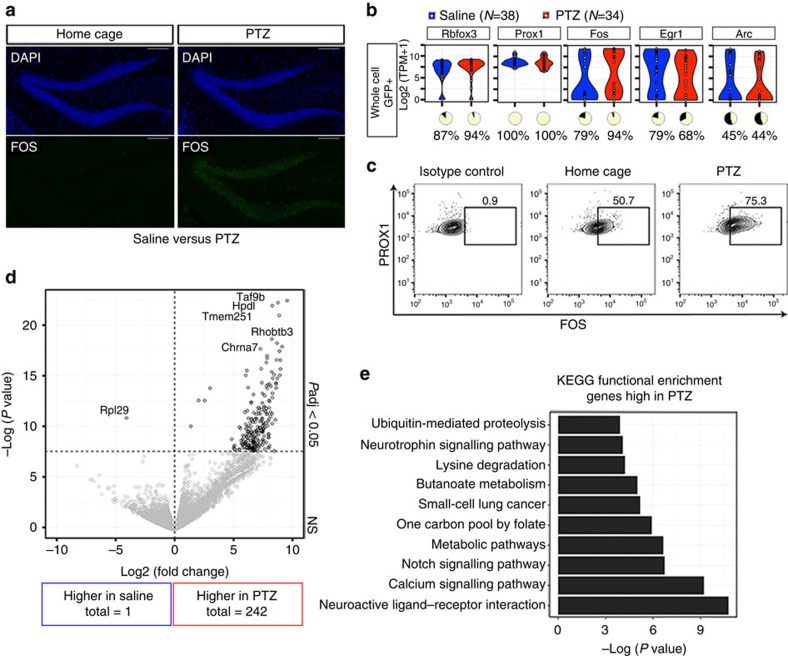
Experience-independent induction of IEG expression in whole cells. (**a**) Confocal images of FOS protein expression in the DG of a HC mouse (left) and a PTZ-treated mouse (right). One hour after PTZ-induced seizure, FOS (green) expression was widespread throughout the granule cell layer, whereas expression under HC conditions was minimal. Scale bar, 100 μm. (**b**) log2(TPM+1) normalized RNA-seq values from dissociated saline-treated (blue, *n*=38) and PTZ-treated (red, *n*=34) whole-cells for neuronal marker *Rbfox3*, DG marker *Prox1*, and IEGs *Fos*, *Egr1* and *Arc*. Pie charts indicate the proportion of samples with detectable gene expression (TPM > 1; yellow). (**c**) Representative FACS plots showing FOS protein expression in PROX1+ live (Zombie -) single whole-cells isolated from the hippocampus of PTZ-treated and HC mice, *n*=2. FOS protein expression was observed in both PTZ-treated and HC animals. (**d**) Gene expression comparison of activated saline-treated and PTZ-treated whole cells. Dots to the left of zero represent genes with higher expression in saline, those to the right represent genes with higher expression in PTZ whole cells. EdgeR was used for differential expression analysis. (**e**) Functional enrichment of genes high in PTZ-treated whole cells using a modified Fisher's exact test (EASE score, DAVID bioinformatics^69^).

As a basis for examining activity, we elicited large-scale neuronal activation with PTZ, a GABA(A) receptor antagonist that induces seizures coupled with IEG expression in DGCs^28^ (Fig. 1a). Mice were either treated with PTZ (*n*=2) or received an injection with saline as a control (*n*=2). One hour after injection, the mice were killed and the hippocampus was dissected. Hippocampi from within groups were pooled for downstream processing. Whole cells were dissociated using papain and subsequently sorted on green fluorescent protein using fluorescence-activated cell sorting (FACS). RNA from 47 individual saline-treated cells and 43 PTZ-treated cells was then amplified for RNA-seq using the Smart-seq2 protocol^2^.

RNA-seq profiles from 38 saline- and 34 PTZ-treated cells passed quality control (QC) (Supplementary Figs 1a-c). An average of 6,272 (+/−1,012) and 6,780 (+/−2,764) genes was detected (detection >1 transcripts per million, TPM) in saline- and PTZ-treated neurons, respectively. The proportion of cells with detectable gene expression of the neuronal marker *Rbfox3* (NEUN) and DGC marker *Prox1* was similar between groups (*Rbfox3*: control=87%, PTZ=94%; *P*=0.52; *Prox1*: control=100%, PTZ=100%; Fig. 1b).

We expected to observe roughly 2% of DGCs activated in the saline-treated whole cells^10^,^18^ and large-scale IEG activation in DGCs of mice injected with PTZ^28^. While it was expected that whole-cell dissociation could moderately increase IEG expression, we were surprised that RNA of the IEGs *Fos*, *Egr1* and *Arc* were detected in an equivalently large proportion and at similar overall expression levels within both control and PTZ-treated neurons: *Fos* (control=79%, PTZ=94%; *P*=0.13), *Egr1* (control=79%, PTZ=68%, *P*=0.41), and *Arc* (control=45%, PTZ=44%; *P*=1; Fig. 1b). This finding indicated that, in these DGCs, IEG mRNA expression was present independent of PTZ treatment.

To determine whether IEG activity observed at the RNA level was also present at the protein level, FOS protein levels were examined in a replicate experiment. The control mouse in this experiment was untreated and remained in the home cage (HC) to exclude the possibility that saline-injection-induced activity before whole-cell preparation. One hour after PTZ injection, the PTZ-treated and HC mice were killed, the hippocampi were dissected, and cells were dissociated with papain. Cells were immunostained for intranuclear PROX1 and FOS and were analysed by FACS. Similar to the observation via RNA-seq, 51% of HC DGCs and 75% of DGCs from PTZ-treated mice were FOS+ at the protein level (Fig. 1c). Therefore, both RNA and protein expression of IEGs were observed independently of the experience of the animal after whole-cell dissociation.

### PTZ induces a transcriptome that is distinct from controls

Activity, as evidenced by *Arc*, *Fos* and *Egr1* expression, was evoked in a subset of both the saline- (15 of 38 cells) and PTZ-treated (11 of 34 cells) animals (Supplementary Fig. 1g). However, we asked whether other gene expression differences persisted that could be attributed to treatment. We examined the differences in gene expression between neurons that had been activated by PTZ in comparison with activation after saline treatment and identified 243 differentially expressed genes (DEGs) after false discovery rate (FDR) correction. Most DEGs were higher in the PTZ-treated group (242 genes), indicating that the neurons activated by PTZ developed a distinct transcriptional profile compared with neurons that were activated independently of treatment (Fig. 1d). Among these DEGs, there was only one previously identified downstream target of c-Fos^29^, *Dap* (1 of 54 genes; hypergeometric *P*=0.36) signifying that common c-Fos activation was not the main factor separating PTZ from saline activation. The genes that were increased after PTZ treatment were significantly enriched in functional pathways such as neuroactive ligand–receptor interaction and calcium signalling (Fig. 1e). These genes included *Chrna7*, a nicotinic acetylcholine receptor that has been previously associated with idiopathic generalized epilepsy^30^,^31^ in humans. These results showed that the neuronal response associated with PTZ treatment induced a unique transcriptional profile that extended beyond the canonical induction of IEGs.

### Immunostaining of nuclei identifies activated DGCs

We next sought to identify a method of single-cell preparation that did not induce IEG expression. An alternative method of dissociating cells that is protease- and heat-free is Dounce homogenization, which is used when isolating individual nuclei from tissue^13^. To determine whether the Dounce method also induced IEG expression, we developed a protocol for nuclei isolation and staining that identified activated DGCs.

**Figure 2:**
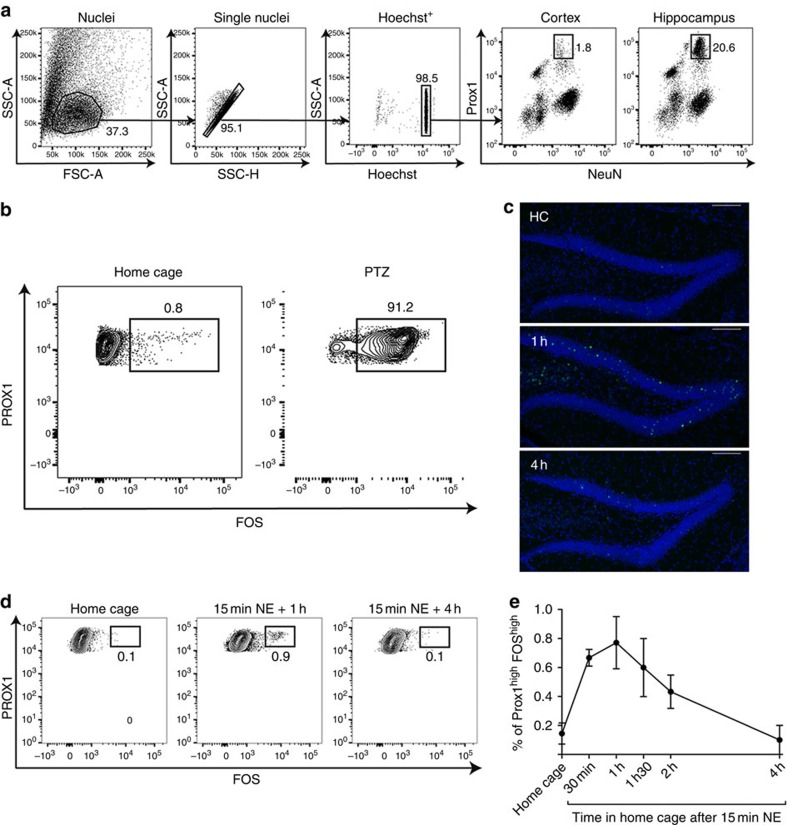
Detection of experience-associated FOS levels with nuclei staining. (**a**) Representative FACS plots showing the gating strategy for the identification of PROX1+NEUN+ DG nuclei in the hippocampus of wild-type mice, *n*=3. Staining in cortex is shown as control. (**b**) Representative FACS plots showing FOS protein expression in PROX1+Hoechst+single-nuclei isolated from the hippocampus of PTZ-treated and HC mice, *n*=4. FOS protein expression was only observed in PTZ-treated animals. (**c**) Confocal images of FOS protein expression in the DG of a mouse either from home cage (HC) (top) or after recovery from a brief exposure to a novel environment (NE; middle and bottom). One hour after a 15-min exploration period in the NE, FOS was sparsely induced in the granule cell layer (middle). Four hours after NE FOS returned to lower levels similar to HC (bottom). Scale bar, 100 μm. (**d**) Representative FACS plots showing FOS protein expression in PROX1+Hoechst+single-nuclei isolated from the hippocampi of animals from HC, 1 h,or 4 h after a 15-min exposure to an NE. (**e**) Percentage of PROX1+FOS^high^ in the hippocampi of animals from HC 0.5 h, 1 h, 1.5 h, 2 h or 4 h after a 15-min exposure to an NE, error bars represent standard deviation from the mean (*N*=4 per time-point).

Nuclei were isolated following a modified version of the previously published procedure^13^,^32^; gradient centrifugation was omitted and an antibody staining protocol was included (Supplementary Fig. 2a). Using flow cytometry, DGC nuclei were identified by size/structure discrimination and Hoechst+ staining (Fig. 2a). An antibody to NEUN was used to label all neurons and PROX1 was used to identify DGCs (NEUN+PROX1+). Staining in the hippocampus was compared with that in nuclei from the cortex, which lacks PROX1 in adult mice (Fig. 2a). The majority of nuclei were PROX1− in both cortex and the hippocampus. In both tissues, a NEUN^-^PROX1^low^ population could be observed. This population also stained positive for the oligodendrocyte marker OLIG2 and was not considered for further analysis. The NEUN+PROX1+ population made up only background levels in the cortex (1.8%) and was observed in 20.6% of the hippocampal nuclei (Fig. 2a). This hippocampal population of NEUN+PROX1+ nuclei was used for downstream analysis.

C-fos binds to Jun-B as part of the AP-1 transcription factor complex that is localized to the nucleus on stimulation^33^, making the staining approach compatible with nuclei dissociation. Since FOS protein was detected in dissociated whole cells independent of PTZ treatment, we first examined the levels of FOS after single-nuclei dissociation from HC and PTZ-treated mice. Importantly, only 0.8% of nuclei from the HC mice had detectable levels of FOS, whereas 91.2% of nuclei from the PTZ-treated mice were FOS+ (Fig. 2b). These results were similar to expectations based on immunostaining of tissue slices.

Next it was important to examine the ability of this nuclei staining approach to detect FOS protein at levels equivalent to those induced by behaviour. Mice exposed to a NE exhibit elevated FOS in DGCs, although with a much smaller proportion of cells compared with PTZ-treated mice^10^ (Fig. 2c). Mice were exposed to NE for 15 min, returned to the HC, and then killed after 0.5, 1, 1.5, 2 or 4 h. To identify the rare population of activated DGCs, nuclei were co-immunostained with PROX1 and FOS. Analysis of FOS by FACS detected similar dynamics as previously reported using immunohistochemistry^15^,^16^(Fig. 2c), with maximum FOS at 1 h after returning to HC (Fig. 2d,e). This finding indicated that the nuclei staining approach was capable of discriminating behaviourally relevant levels of FOS protein.

### Synthetic pool of single nuclei reflect bulk DG

To determine the reliability of single-nuclei signatures, we evaluated how synthetically pooling single-nuclei samples compared with bulk DG. DGC nuclei were identified by co-immunostaining for the transcription factors NEUN and PROX1 and gene expression was analysed for 23 NEUN+PROX1+ nuclei dissociated from HC dentate gyrus that passed QC (Supplementary Figs 2b,c). Since RNA isolated from the nucleus represents a portion of the total RNA within a cell, we first examined expression in DGC nuclei with respect to overall gene complexity and overlap with bulk DG nuclei derived from 500 sorted DG nuclei.

To determine the overlap of expression between single DGC nuclei and bulk DG, individual NEUN+PROX1+ nuclei were ‘synthetically' pooled with increasing numbers of nuclei per pool. The overlap of genes detected in the synthetic pool and bulk tissue was calculated across multiple sampling events. Pooling all 23 nuclei resulted in a maximum of 72% overlap of genes (Fisher's exact test *P*<2.2e−16) with a TPM>1, with a correlation between single and bulk of 0.58 (Pearson's *P*<2.2e−16) (Supplementary Figs 2e,f). This overlap was similar to previous observations from whole lymphoblastoid cells^34^.

### SnRNA-seq detects IEGs concordant to behavioural experience

We next sought to determine whether IEG RNA expression was detectable within single nuclei with high sensitivity using exposure to NE to activate DGCs. For context exposure, mice remained in the HC or were placed into NE for 15 min. NE mice were returned to the HC for 1 h before both HC and NE groups were killed; brains were collected for nuclei dissociation and immunostained for PROX1 and FOS. FACS analysis identified a small population of neurons (∼1%) that had substantially higher FOS protein levels than the HC controls (Fig. 2d).

Nuclei from NE DGCs were directly sorted into a 384-well plate and amplified using the Smart-seq2 protocol^35^. Gene expression was analysed for 36 PROX1+FOS+ and 43 PROX1+FOS− nuclei that passed QC (Supplementary Fig. 3a–c). Gene detection between NE nuclei groups was highly similar, with an average of 6,390 (±1,200) and 6281 (±1,548) genes detected in FOS+ and FOS− nuclei, respectively (Supplementary Fig. 3c).

**Figure 3:**
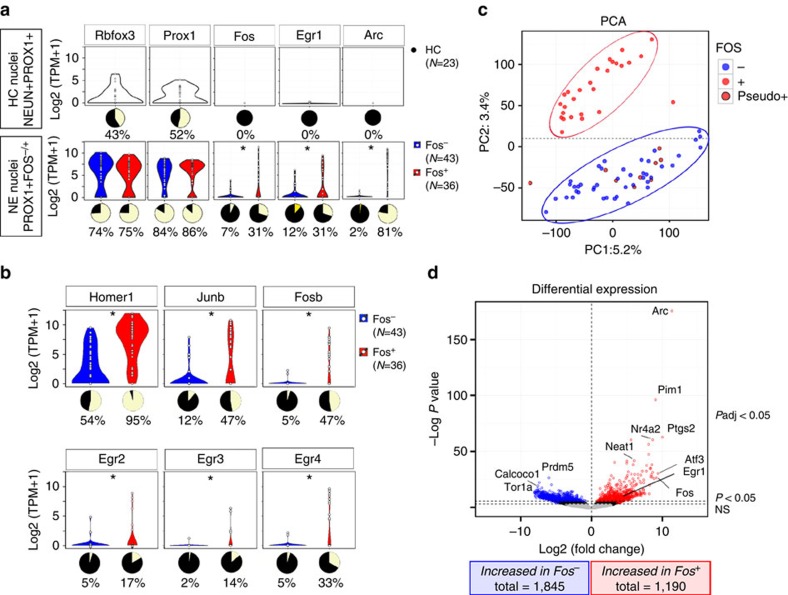
IEG RNA expression in single DGC nuclei is associated with animal experience. (**a**) log2(TPM+1) Normalized RNA-seq values from HC NEUN+PROX1+ (top, *n*=23), NE PROX1+FOS− (bottom, *n*=43), and NE PROX1+FOS+ (bottom, *n*=36) single-nuclei. NE FOS+ nuclei (red) exhibited higher levels of IEG expression than both NE FOS− (blue) and HC nuclei (white). Stars indicate statistically significant differences in expression using edgeR after multiple-testing correction (fdrtool; R). Pie charts indicate the proportion of nuclei with detectable gene expression (yellow=detected). (**b**) IEG expression in nuclei after exposure to NE. (**c**) Principal components analysis (PCA) of the full transcriptome for NE nuclei. pseudo-FOS+ cells in red with black outline. (**d**) Differential expression results for all genes between FOS+ and FOS− nuclei excluding pseudo-FOS+ nuclei. Genes expressed to a higher level in FOS+ nuclei are in red and genes expressed higher in FOS− nuclei are in blue.

Although overall detection efficiencies were slightly lower in nuclei from HC, both HC and NE nuclei exhibited expression of the neural marker *Rbfox3* and DGC marker *Prox1*. *Rbfox3* was detected in 43% of the HC DGC nuclei and *Prox1* in 52% (Fig. 3a—top). Importantly, the expression of *Rbfox3* and *Prox1* were independent of FOS staining in nuclei from the NE condition (*Rbfox3:* FOS−: 74%, FOS+: 75%; *P*=1) (*Prox1*: FOS−: 84%, FOS+: 86%; *P*=0.94) (Fig. 3a-bottom). Based on immunostaining, we expected to observe only low levels of IEG expression in both HC and NE FOS− nuclei. Indeed, in nuclei from the HC mouse, *Fos*, *Arc* and *Egr1* were not detected (detection=1 TPM; Fig. 3a—top). To confirm the low IEG expression in HC nuclei, we ran a separate experiment using single-cell/nuclei quantitative PCR (qPCR) as an assay. All single nuclei (*n*=39) and whole cells (*n*=13) examined expressed equivalent levels of *Rbfox3* and *Prox1*. However, 100% of whole-cells expressed *Fos* in comparison with only 2% of the single nuclei (Supplementary Fig. 2g).

FOS− nuclei from the NE mouse also exhibited relatively low levels of IEG expression, as expected based on immunostaining (Fig. 3a—bottom). Conversely, FOS+ nuclei from the NE mouse exhibited significantly higher levels of the IEGs *Fos*, *Egr1, and Arc* (*Fos*: FOS−=7%, FOS+=31%; *P*<0.02; *Egr1*: FOS−=12%, FOS+=31%; *P*=0.09; *Arc*: FOS−=2%, FOS+=81%; *P*<1.9e−11) (Fig. 3a—bottom). Other IEGs, including *Homer1*, *Junb*, *Fosb*, *Egr2*, *Egr3* and *Egr4*, also consistently showed higher expression in FOS+ nuclei (Fig. 3b), indicating that IEG expression was detectable in single nuclei after experience-induced activation. Taken together, these results indicated that single-nuclei preparations were capable of distinguishing changes in IEG expression due to experience without eliciting additional activity-related gene expression from the preparation method.

### Large-scale transcriptional response to activity in DGCs

**Figure 4:**
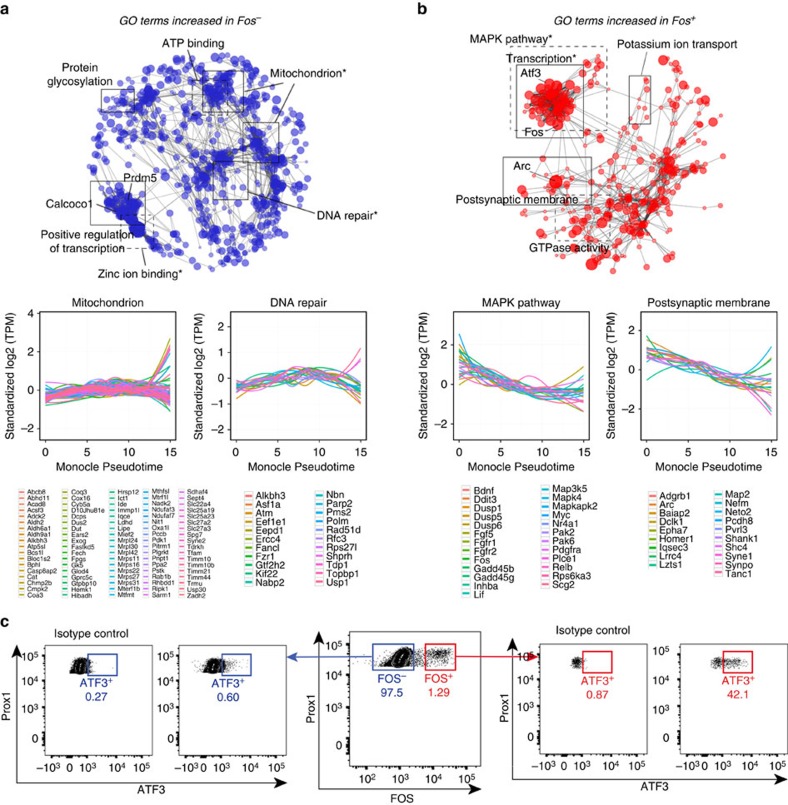
Gene ontology for FOS− and FOS+ nuclei. Clustering based on Gene Ontology terms for differentially expressed genes either higher in FOS− nuclei (**a**) or FOS+ nuclei (**b**). Two categories are plotted below as a function of pseudotime using Monocle. (**c**) Representative FACS plots showing expression of ATF3+ in single-nuclei isolated from the hippocampus of a wild-type mouse exposed 15 min to an NE and killed 1 h later. Right panel shows expression of ATF protein in PROX1+FOS+ nuclei and the left panel shows ATF in PROX1+FOS− nuclei. 42.1% of PROX1+FOS+ nuclei co-express ATF3 as indicated by the red gate. Asterisks indicate significant functional enrichment after multiple-testing correction using DAVID.

With IEGs expressed preferentially in the FOS+ nuclei of the NE condition, we next sought to examine the transcriptional profile of Fos-activated DGCs in further detail. Principal components analysis split the samples into two distinct groups that were associated with Fos staining (F-test PC1: *P*<0.05; F-test PC2: *P*<2.5e−14; Fig. 3c). Interestingly, there were nine nuclei that had high FOS protein expression but clustered with FOS− nuclei based on gene expression. These nuclei will hereafter be referred to as pseudo-FOS+ nuclei. Similar to FOS− nuclei, these pseudo-FOS+ nuclei showed low expression of *Arc*, *Fos* and *Egr1* RNA (Supplementary Fig. 3e). Importantly, classification as pseudo-FOS+ was not associated with sample read depth (F-test *P*=0.24), indicating that low read-depth was not a likely cause of the observed differences in expression. Since these nuclei represented a population that was distinct from both FOS+ and FOS− nuclei, pseudo-FOS+ nuclei were filtered out from the first analysis aimed at determining bulk differences between FOS+ and FOS− cells. The remaining nuclei were examined as biological replicates. Differential expression analysis revealed a total of 3,035 DEGs after FDR (Fig. 3d, Supplementary Data 1), indicating a large-scale transcriptional shift due to exposure to NE. Approximately half of the DEGs were higher in FOS− nuclei (1,845 genes) compared with FOS+ (1,190 genes). Furthermore, the most significant differences were detected as higher in the FOS+ condition. As expected, many of the genes increased in FOS+ nuclei have been previously shown to be downstream targets of FOS^29^ (19 of 54; hypergeometric *P*<2.3e−9), indicating that the expected gene expression changes were detected by the single-nuclei protocol. Furthermore, genes involved in the MAPK pathway (for example, *Mapk4* and *Dusp1*) and associated with postsynaptic density (for example, *Homer1*, *Arc* and *Synpo*) were increased in FOS+ nuclei (Fig. 4b). It was also noted that the B2 family of SINE retrotransposons was increased in FOS+ nuclei (Supplementary Fig. 3f). In FOS− nuclei, genes that were associated with mitochondrial function (for example, *Nos1* and *Ndufaf3*) as well as genes associated with DNA damage (for example, *Topbp1* and *Tdp1*; Fig. 4a) were increased. Interestingly, recent findings have shown that rapid Fos activation can be initiated by Topoisomerase II-induced damage^36^. The DEG Topbp1 is a DNA damage checkpoint protein^37^ that binds topoisomerase II (ref. 38), and Tdp1 repairs topoisomerases I and II DNA damage^39^, indicating that these cells may be undergoing repair due to topoisomerase damage.

Further characterization of DEGs revealed that distinct subsets of transcriptional regulators were upregulated specifically in FOS+ (for example, *Atf3* and *Sertad1*) or FOS− (for example, *Calcoco1* and *Esrra*) nuclei (Supplementary Table 1). Since single-nuclei preparation enables direct staining of transcription factors, we assayed the top transcription factor that was induced in FOS+ nuclei. *Atf3* is a transcription factor that is normally expressed at relatively low levels in neurons and increases on exposure to injury^40^ as well as enriched environment exposure^41^. Similar to snRNA-seq, ATF3 protein was enriched in PROX1+FOS+ nuclei (42.1%) compared with PROX1+FOS− (0.60%; Fig. 4c).

Altogether, these results show that snRNA-seq identified large-scale transcriptional changes in DG neurons after a short, 15-min exposure to NE.

### Heterogeneous gene expression in DG nuclei with NE exposure

The vast majority of IEG experiments do not utilize temporal information, unlike electrophysiology or calcium imaging. However, tools have been developed such as cellular compartment analysis of temporal activity by fluorescence *in situ* hybridization^42^ and transgenic mice^10^,^43^ to evaluate two time points of activity. Recent single-cell analysis methods^44^,^45^ have utilized high-dimensional data to re-construct developmental pathways through pseudotime. We applied the Monocle algorithm to our snRNA-seq data from FOS+ and FOS− nuclei to determine a continuum of pseudotemporal activity patterns in experience-activated neurons.

**Figure 5:**
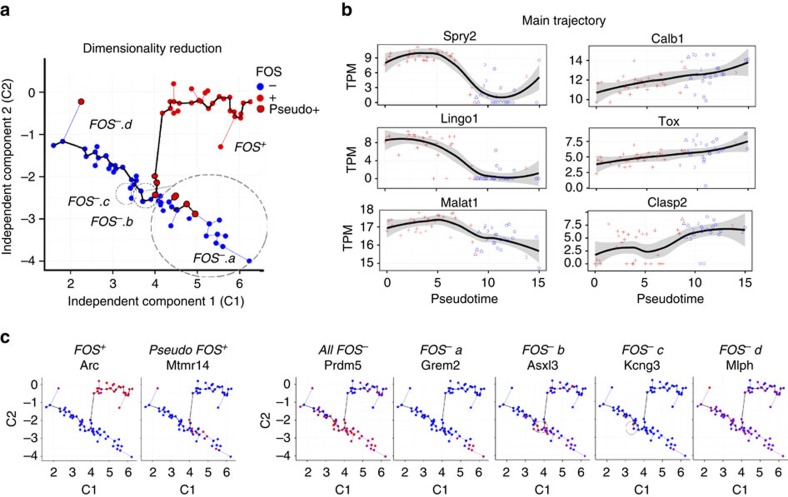
NE nuclei exist along a continuum of transcriptional states. (**a**) Independent components based on analysis using Monocle with individual nuclei coloured by FOS protein staining. Pseudo-FOS+ nuclei (red with black outline) segregate towards the border of FOS+ (red) and FOS− (blue) nuclei. (**b**) The top genes associated with pseudotime exhibit continuous patterns of expression linking FOS+ (red) and FOS− nuclei (blue). (**c**) Expression based on cluster, high expression=red, low expression=blue.

Nuclei were ordered by their transcriptional similarity using the Monocle algorithm^45^ with a supervised approach by first filtering for DEGs based on the above FOS+/FOS− analysis. Similar to the unsupervised principal components analysis (Fig. 3b), the independent components separated NE nuclei into two main groups (Fig. 5a; Supplementary Fig. 4a) that were highly associated with FOS protein staining. Monocle assigned the nuclei into five clusters that we have labelled as FOS+ or FOS− *a*-*d*.

Pseudo-FOS+ nuclei resided largely at the junction between FOS+ and FOS− clusters (Fig. 5a, red dots with black outline), indicating that pseudo-FOS+ nuclei were transcriptionally similar to FOS− nuclei despite being stained positive for FOS protein. On further examination, there were very few genes (raw*P*<0.05: 845 genes; *P*adj<0.05: 0 genes) that were different between the neighbouring cluster of FOS− nuclei (FOS− *b*) and pseudo-FOS+ nuclei. However, similar to FOS− nuclei there were many genes increased in pseudo-FOS+ nuclei compared with FOS+ (*P*adj<0.05: 713 genes) (Supplementary Fig. 4c). Taken together, these findings suggest that pseudo-FOS+ cells likely do not represent a distinct state of cells, but rather cells at an intermediate stage in the progression of FOS-associated activity.

The trajectory of activity captured in NE nuclei can be viewed from the main trajectory identified with Monocole (Fig. 5a, thick line) that was significantly associated with FOS stain (F-test *P*<9.59e−09). To assess linear changes in gene expression, TPM expression was regressed against pseudotemporal ordering. To reduce effects of outlier expression that may exist within clusters FOS- *a* and FOS- *c*, only samples along the main trajectory were included in the analysis. A total of 641 genes were significantly associated with pseudotime (Fig. 5b). For example, *Clasp2* which is important in neuronal morphogenesis^46^, continuously increased with pseudotime indicating that a continuum of transcriptional states existed within a single DG after exposure to NE. The transcriptional dynamics that separated clusters FOS-*a* and FOS-*c* off from the main trajectory were examined by calculating differences in expression between the individual cluster and all remaining samples (Fig. 5a). Cluster FOS- *a* was isolated by expression of *Grem2*, a DAN family BMP antagonist, as well as genes enriched for lysine degradation (*P*adj<0.03; for example, *Ogdhl* and *Plod1*). Cluster FOS-*c* did not associate with a distinct functional category but exhibited increased expression of many genes including the potassium channel gene Kcng3 (Fig. 5c).

We next asked whether neuronal heterogeneity was associated with the expression of genes upstream of FOS. CREB has been proposed to serve such a role; experiments altering CREB in the amygdala influenced the probability that neurons were recruited into a memory trace^47^. However, experiments determining whether endogenous levels of CREB or other genes influence activation have not been performed. To address this question, we evaluated the signalling pathway that translates neuronal activity to *Fos* induction within the Monocle clusters. While *Fos* can be turned on by a number of different pathways from development or non-physiological stimuli^48^,^49^, the molecular cascade that leads to activity-induced FOS expression in the brains of behaving mice is by a well-known pathway that is initiated by calcium influx through NMDA receptors and voltage-sensitive calcium channels^50^,^51^,^52^. Calcium influx then triggers ERK/MAPK, which in turn stimulates phosphorylation of ELK1-serum response factor (SRF) and RSK phosphorylation of CREB^53^. Phosphorylated-CREB then binds to the CRE-element of the *Fos* promoter to mediate its induction^54^.

We first analysed the expression of membrane-bound receptor channels that are known to influence neuronal activity and calcium influx. Genes encoding glutamate receptors including NMDA receptor subunits (*Grin1, Grin2a* and *Grin2b*) and AMPA receptor subunits (*Gria1-4*), were highly expressed and not significantly different between NE FOS− and NE FOS+ nuclei. GABA receptors are well known to mediate inhibition. GABA-A receptor subunits (*Gabra1, Gabra2* and *Gabrb*) were also expressed similarly between the groups. Likewise, we analysed the expression of voltage-sensitive calcium channels that are the primary mediators of depolarization-induced calcium entry^55^. High-(ref. 56) and low-voltage-activated^57^ calcium channels are known to be expressed on DG neurons. However, genes encoding alpha subunits for L-, N/Q and T-type calcium channels and from the Cav1, Cav2 and Cav3 families (*Cacna1a-i*) were not expressed at different levels in our NE nuclei data set.

We then turned our attention to the underlying molecular cascade that transduces calcium activity into *Fos* expression, the ERK/MAPK pathway. Examination of the NE nuclei shows no significant differences for *Mapk3* (encoding ERK1), *Mapk1* (encoding ERK2), *Elk1*, *Srf* or *Creb1*. Most subunits of the RSK protein were not differentially expressed. However, one subunit, *Rps6ka3*, showed trending significance with the Monocle FOS+ subgroup (*P* value=3.0e−4, *P*adj _group FOS+_=0.08, Supplementary Fig. 4d). Importantly, it would be expected that this gene would be expressed in FOS− cells, although at a lower level, since Rps6ka3 lies upstream of c-Fos activation. Higher levels of *Rps6ka3* would increase the likelihood of RSK phosphorylation of CREB and downstream activation of *Fos*.

In summary, Monocle analysis of snRNA-seq established a continuum of heterogeneous activation states in IEG-expressing cells, increasing the temporal resolution that can be provided by gene expression.

## Discussion

Single-cell profiling of neural tissue has become an increasing area of interest; however, caution must be exercised when using this information to study patterns of activity-related expression. We found that, in our hands, papain dissociation of whole-cells from the DG elicited IEG expression independent of the condition of the animal. Although we have chosen to focus on the DG, the methodology described here to link c-fos expression and transcriptome analysis could be applied throughout the brain. Decades of work have demonstrated that, in any region where current understanding of anatomical, morphological, and molecular markers is insufficient to predict whether an individual cell will respond to a particular stimulus, IEG-based methods are a valuable tool for identifying subpopulations. C-fos-based strategies have been critical for understanding the functional organization of areas such as the amygdala^58^, hypothalamus^59^,^60^ , and somatosensory cortex^61^, where subpopulations of cells with differential involvement in sensory perception and behaviour are anatomically intermingled.

The assessment of a bulk IEG-labelled population has been performed in cocaine-induced activity of the striatum via flow sorting^62^. To our knowledge, this is the first reported case of comprehensive signatures of activation from individual cells. IEGs that have been previously associated with activity were readily identified using snRNA-seq, and we have also demonstrated that activated neurons exhibited large-scale gene expression changes beyond these canonical IEGs. Interestingly, many of the genes upstream of the activation of FOS were similarly expressed between FOS+ and FOS− neurons, supporting future work into examining the activation state of a neuron via protein modifications. The joint analysis of FOS+ and FOS− nuclei after exposure to NE allowed us to construct a pseudotemporal activation pattern using an algorithm that was previously applied to developing myoblasts. This pseudotemporal ordering revealed a continuum of activity with overlapping gene expression patterns between a set of FOS+ (pseudo-FOS+) and FOS− nuclei, as well as gene expression profiles in a subset of FOS− nuclei that were indicative of recent activity.

The ability to combine the identification of single activated cells with robust transcriptome analysis will continue to provide a wealth of novel insights into the mechanisms that govern how individual cells contribute to such circuits. Together, these findings demonstrate that snRNA-seq is a robust technique for examining such heterogeneous transcriptional profiles from activated neurons.

## Methods

### Animals and treatment

All animal procedures were approved by the Institutional Animal Care and Use Committee of The Salk Institute for Biological Studies. Wild-type female C57BL/6 mice (8 weeks old) were purchased from Harlan (San Diego) and housed under standard 12-h light/dark cycles with free access to food and water. *Prox1*-EGFP mice were obtained from the Mutant Mouse Regional Resource Center. Dimensions of the NE cage were 54" × 34" base, 12" height. The NE included huts and tunnels that the animal was not previously exposed to. For PTZ treatment, mice were injected with 50 mg kg^−1^ fresh PTZ dissolved in saline (intraperitoneal; at a volume of 20 ml kg^−1^ body weight). The control mouse was injected with 400 μl of saline. Mice were killed 1 h after injection. The mice exposed to PTZ exhibited seizure-like symptoms beginning 5 min after PTZ injection and continuing for 10 min. Saline-injected mice did not exhibit any seizure symptoms.

### Whole-cell and nuclei dissociation

To dissociate whole cells, Worthington Biochemical Corporation's Papain Dissociation System was used according to the manufacturer instructions (Worthington, cat# LK003150). Briefly, mice were anaesthetized with ketamine/xylazine (100 mg kg^−1^, 20 mg kg^−1^, respectively) and killed, and their brains were removed. Cortex, hippocampus or DG was carefully excised and minced slightly. The tissue was incubated in a papain solution (20 units per ml papain, 0.005% DNAse) at 37 °C for 1–1.5 h with gentle agitation, followed by gentle trituration. Dissociated cells were pelleted and resuspended in ovomucoid inhibitor–albumin medium. Centrifugation of a single-step discontinuous density gradient separated intact cells. The pellet, containing the intact cells, was resuspended in PBS and immunostaining was performed. For nuclei dissociation, tissue was collected as described for whole cells. The nuclei dissociation protocol is similar to the previously described protocol^13^ with some modifications. Cortex, hippocampus or DG was carefully excised and immediately placed into a nuclei isolation medium (sucrose 0.25 M, KCl 25 mM, MgCl_2_ 5 mM, TrisCl 10 mM, dithiothreitol, 0.1 % Triton). Tissue was Dounce homogenized, allowing for mechanical separation of nuclei from cells. The nucleic acid stain Hoechst 33342 (5 μM, Life Technologies) was included in the media to facilitate visualization of the nuclei. Samples were washed, resuspended in nuclei storage buffer (sucrose, MgCl_2_ 5 mM, and TrisCl 10 mM) and filtered. Solutions and samples were kept cold throughout the protocol.

### Slice immunohistochemistry

Mice were exposed to 15 min of NE or PTZ or remained in the HC as previously described. One hour later, they were deeply anaesthetized with ketamine/xylazine and transcardially perfused with 0.9% NaCl followed by 4% paraformaldehyde. Brains were removed, post-fixed overnight and transferred to 30% sucrose for 2 days. Forty-micrometre coronal slices spanning the anterior-posterior extent of the hippocampus were sectioned on a microtome and stored at −20 °C until staining. Immunostaining for FOS was performed with a goat anti-FOS primary antibody (sc-52-G, Santa Cruz, 1:250) and donkey anti-goat AF488 secondary (Jackson ImmunoResearch, 1:250). Nuclei were visualized using DAPI (1.0 μl ml^−1^). Sections were mounted on #1.5 glass coverslips using PVA-DABCO mounting media. Confocal images were acquired on a Zeiss LSM 780 laser scanning confocal microscope using a × 20 objective. Z-stack images with a 2.3-μm interval were taken of the DGC layer using two horizontal tiles to fit the entire DGC into the image. Tiles were stitched using ZEN2011 software, and maximum intensity projections were created for figure display.

### Flow cytometry

To immunostain, dissociated nuclei were incubated with mouse IgG2b anti-PROX1 (4G10, EMD Millipore, 1/400), goat anti-FOS (sc-52-G, Santa Cruz, 1/400). Samples were then stained with the following secondary antibodies: donkey anti-mouse-Cy3 (Jackson ImmunoResearch, 1/500), donkey anti-goat-AF647 (Jackson ImmunoResearch, 1/500). Centrifugation of secondary-stained samples was followed by resuspension in mouse IgG1 anti-NEUN-AF488 (A60, EMD Millipore) in the presence of DNA dye Hoechst 33342. Dissociated whole cells were first stained with Zombie UV Fixable Viability kit (Biolegend); cells were then fixed and permeabilized with Nuclear Factor Fixation and Permeabilization Buffer Set (Biolegend) according to manufacturer's protocol prior to staining with the same antibodies as nuclei. Data from labelled samples were acquired using a BD LSRII cytometer (BD Biosciences) followed by analysis using FlowJo software (Tree Star). For both flow analysis and FACS, Zombie dye-negative cells or Hoechst-positive nuclei were gated first, followed by exclusion of debris using forward and side scatter pulse area parameters (FSC-A and SSC-A), exclusion of aggregates using pulse width (FSC-W and SSC-W), before gating populations based on PROX1, FOS, and NEUN fluorescence. For collection before amplification, a BD Influx sorter was used to isolate cells and nuclei, with PBS for sheath fluid (100-μm nozzle was used for cells with sheath pressure set to 16.5PSI; nuclei were sorted with an 85-μm nozzle at 22.5 PSI sheath pressure). Single cell/nuclei were directly deposited into individual wells containing 2 μl lysis buffer in 384-well plate format. For single cell/nuclei sorting, Single Cell (1 drop) sort mode was selected for counting accuracy. Fine mechanical alignment of the sorter's plate module was facilitated by sorting 20 10-μm fluorescent beads onto the surface of transparent plate sealer (adhered to a 384-well plate), making positional adjustments as necessary; 20 beads were then sorted directly into the bottom of various well positions throughout an empty plate to provide visual confirmation of counting and targeting precision.

### Single-cell/-nuclei amplifications

The HC NEUN+PROX1+ single-nuclei were processed using the Fluidigm C1 system, a commercial platform that utilizes microfluidics to perform individual cell capture and reaction chemistry. Stained nuclei were loaded onto the chip and imaged at each capture site on a confocal microscope (Zeiss). Capture sites were scored for NEUN and PROX1 staining expression off-line. Processing of whole cells and NE nuclei followed the Smart-seq2 protocol^2^. NE nuclei from the same isolation pool were prepared in wells of different amplification conditions. Equal proportions of FOS+ and FOS− nuclei were prepared using TSO concentration of 1 μM or 10 μM starting concentration and ERCC dilution of 1:200 or 1:2,000. Differences in mm10-mapped read number and number of genes did not vary between conditions and therefore we combined samples for analysis. In separate experiments, single-cell/nuclei qPCR was performed on the C1 by first utilizing specific target amplification for genes of interest. Samples were then run on the Fluidigm Biomark system for qPCR measurements.

### RNA extraction from bulk samples

Nuclei from DG were dissociated and stained as described above. Nuclei were then bulk collected into a tube containing lysis buffer and amplified using the SMARTer protocol (Clontech) with 14 cycles of PCR.

### Library preparation and sequencing

The Nextera XT kit was used for sample preparation following all transcriptome amplifications. Single-nuclei and amplified bulk samples were measured by Picogreen (LifeTech) and normalized so that samples were at a concentration of 0.3 ng μl^−1^. Samples were first subjected to a tagmentation reaction, indexed, and PCR amplified. Libraries were then mixed in 40-sample pools and purified with magnetic beads (AMPure). QC checks for the library preparations included electrophoresis (Agilent Technologies 2100 Bioanalyzer) or library quantification (KAPA Biosystems). Samples were sequenced in the Salk Institute Next Generation Sequencing Core on an Illumina HiSeq 2500 high-throughput sequencing system. Libraries from saline- and PTZ-treated whole cells as well as the HC NEUN+PROX1+ nuclei were sequenced with paired-end 100-bp reads. NE nuclei libraries were sequenced with single-end 50-bp reads.

### Alignment and generation of data matrices

Reads were trimmed using Solexa-QA++ dynamic trim and were then aligned to the mm10 (GRCm38) reference genome with Ensembl gene annotation or to the ERCC reference using RSEM (bowtie). TPM values calculated by RSEM were log2+1 transformed. Gene names were converted to official gene symbols using biomaRt in R.

### Sample exclusion

Samples were kept for downstream analysis if they met the following criteria: (i) the total number of reads uniquely aligned to the mouse genome was greater than 50,000, (ii) ERCC TPM estimates correlated with the expected concentration values (*α*=0.05), and (iii) total gene count was greater than mean—2σ.

### Statistical analyses

Synthetic pooling of nuclei was achieved by repetitively sampling nuclei, without repetition, and detecting genes with TPM>0 in at least one nucleus. Twenty-five repetitions were used as long as every repeated sample contained a unique set. If the number of unique sets was <25, then the repetition size was reduced accordingly. In initial analyses of NE nuclei, analysis was performed blinded to FOS immunostain group. Significance estimates of differences in gene expression were calculated from RSEM's expected counts using edgeR^63^ and corrected for multiple testing using the fdrtool package in R. Proportion differences were calculated using a test of equal proportions in R (prop.test) with Yates' correction. Regression analyses comparing the clusters identified with Monocle was performed by regressing log2(TPM+1) on the cluster designation (as a factor) for each gene.

### Power analysis

The minimum sample size required to determine significance between groups was calculated a priori using the t-test family in G × Power version 3.1 (ref. 64). The parameters were as follows: effect size=1, *α*=0.05, power=0.95, allocation ratio (N2/N1)=1.14 (as per our actual data, n_Fos+_/n_FOS−_). The calculation yielded the requirement of a minimum of 30 and 26 samples.

### Retrotransposon expression

Total retrotransposon expression was performed by first determining all reads that aligned to the rodent (rodrep.ref) RepeatMasker reference library^65^ using the NCBI Blast 2.2.29 algorithm^66^. Retrotransposon sequences are present in Pol-II gene products as well as RNA products driven from expression of the *bona fide* retrotransposon element. Therefore, in order to calculate a conservative estimate of retrotransposon expression we filtered for reads that were putatively expressed directly from the retrotransposon. This was achieved by filtering for reads where the template-switch oligo sequence was directly adjacent to the beginning of the retrotransposon consensus sequence. These counts were normalized by the total counts aligned to the respective retrotransposon consensus within the respective sample (promoter-normalized count).

### Pseudotemporal ordering

The genes used to calculate pseudotemporal ordering were filtered from the EdgeR results of the comparison between PROX1+FOS+ and PROX1+FOS− nuclei. Genes were required to be differentially expressed between the two groups given an adjusted *P* value<0.01. This gene set was then used for pseudotemporal ordering using Monocle^45^. To determine the robustness of pseudotemporal ordering, the ordering algorithm was repeated for each permutation, where one sample was excluded per permutation.

### ENCODE annotations and gene ontology networks

ENCODE annotations^67^ were used to calculate the proportion of protein-coding genes. To create gene ontology networks, for each gene, gene ontology (GO) terms were extracted using biomaRt. A matrix {0,1} was constructed with a row per gene and a column per GO term. A value of 1 in a cell indicated that the gene was annotated with the respective go term. For each GO term, the number of parents was calculated using GO.db in R; this value was used as a weight for the respective column. The cosine similarity was then calculated between all genes and the resulting matrix was filtered to contain connections between genes where the cosine similarity was >0.5. The inverse of this network (1—cosine similarity) was then plotted in Cytoscape 3.1 (ref. 68).

## Additional information

**Accession codes:** NCBI GEO Accession Number: GSE77067.

**How to cite this article:** Lacar, B. *et al*. Nuclear RNA-seq of single neurons reveals molecular signatures of activation. *Nat. Commun.* 7:11022 doi: 10.1038/ncomms11022 (2016).

## Supplementary Material

Supplementary Information Supplementary Figures 1-4 and Supplementary Table 1

Supplementary dataset Supplementary dataset 1
