# Corrigendum: Nuclear RNA-seq of single neurons reveals molecular signatures of activation

**DOI:** 10.1038/ncomms12020

**Published:** 2016-06-14

**Authors:** Benjamin Lacar, Sara B. Linker, Baptiste N. Jaeger, Suguna Rani Krishnaswami, Jerika J. Barron, Martijn J. E. Kelder, Sarah L. Parylak, Apuã C. M. Paquola, Pratap Venepally, Mark Novotny, Carolyn O'Connor, Conor Fitzpatrick, Jennifer A. Erwin, Jonathan Y. Hsu, David Husband, Michael J. McConnell, Roger Lasken, Fred H. Gage

*Nature Communications* 7: Article number:1102210.1038/ncomms11022 (2016); Published 04192016; Updated 06142016

In the original version of this Article, the middle names or initials of the authors Suguna Rani Krishnaswami, Jerika J. Barron, Martijn J.E. Kelder, Sarah L. Parylak, Apuã C.M. Paquola and Jennifer A. Erwin were omitted from the author information. In addition, financial support for Benjamin Lacar from the UCSD Temporal Dynamics of Learning Center was not acknowledged. These errors have now been corrected in both the PDF and HTML versions of the Article.

