# Corrigendum: Nuclear RNA-seq of single neurons reveals molecular signatures of activation

**DOI:** 10.1038/ncomms15047

**Published:** 2017-03-17

**Authors:** Benjamin Lacar, Sara B. Linker, Baptiste N. Jaeger, Suguna Rani Krishnaswami, Jerika J. Barron, Martijn J. E. Kelder, Sarah L Parylak, Apuã C. M. Paquola, Pratap Venepally, Mark Novotny, Carolyn O'Connor, Conor Fitzpatrick, Jennifer A. Erwin, Jonathan Y. Hsu, David Husband, Michael J. McConnell, Roger Lasken, Fred H. Gage

Nature Communications 7: Article number: 11022; DOI: 10.1038/ncomms11022 (2016); Published 04 19 2016; Updated 03 17 2017

An incorrect version of Supplementary Data 1, in which normalized counts were analysed instead of raw counts, resulting in a smaller number of differentially expressed genes, was inadvertently published with this article. This version was not used in any of the analyses presented in the paper. The HTML has now been updated to include the correct version of Supplementary Data 1.

## 

