# Supplementary material for: Nuclear RNA-seq of single neurons reveals molecular signatures of activation
Source: Nat Commun. 2016 Apr 19;7:11022. doi: 10.1038/ncomms11022 (PMC4838832; doi:10.1038/ncomms11022)
Supplement: Supplementary Information — Supplementary Figures 1-4 and Supplementary Table 1 [file ncomms11022-s1.pdf]

SUPPLEMENTARY DATA

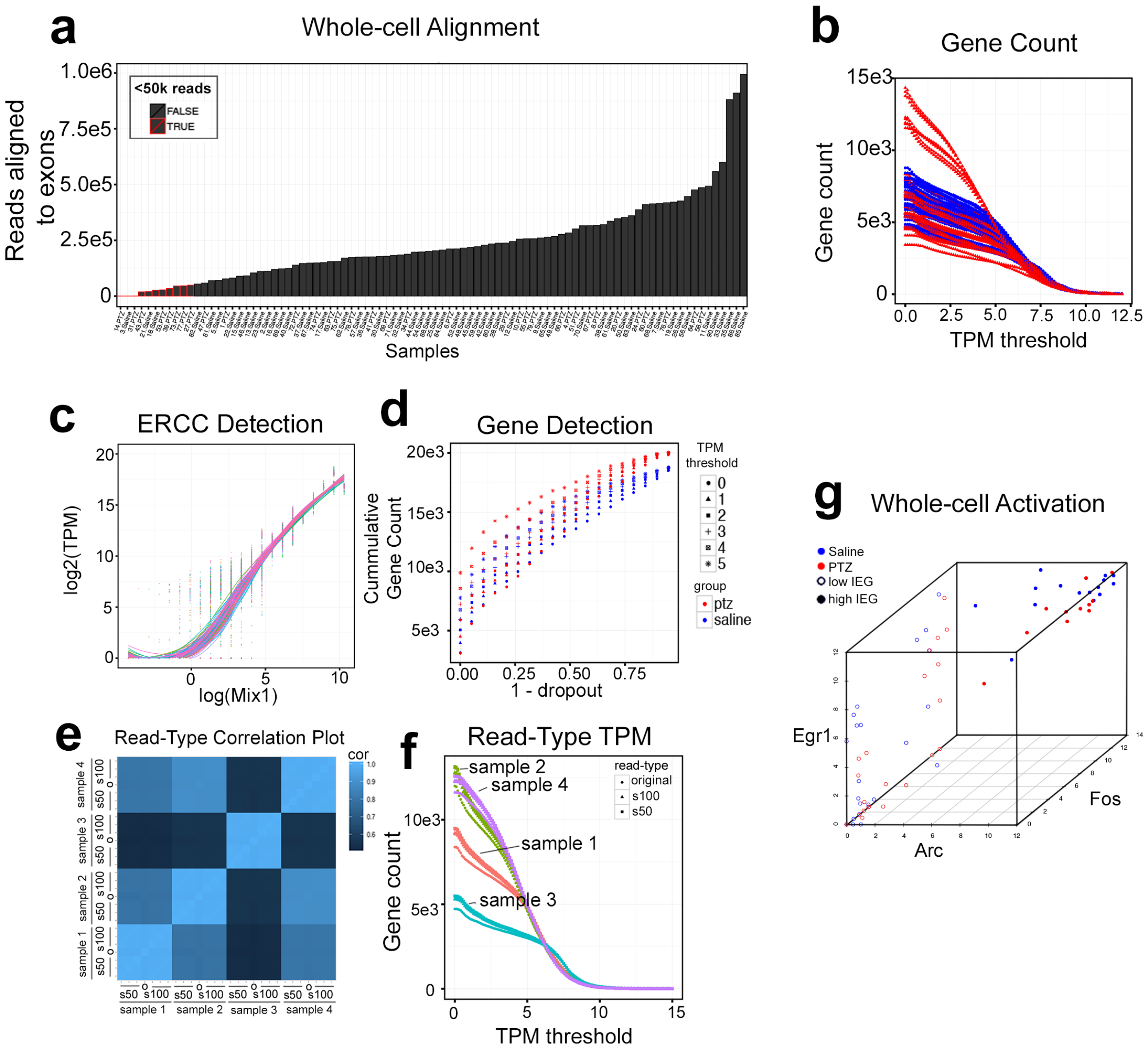

Supplementary Figure 1. Whole-cell RNA-seq quality measures

a) Number of aligned reads for each whole-cell sample. Samples excluded from further analysis are colored in red. b) Gene count as a function of TPM threshold. Red= PTZ-treated whole-cells, blue= saline-treated whole-cells. Each line represents an individual sample. c) Detection of spiked-in ERCC molecules against the original concentration of Mix1. Each line is a smoothed function over each sample. d) Gene count as a function of the total number of samples with detectable expression of a given

gene (1 – dropout). Detectable expression was defined as TPM > x where x is denoted by separate shapes. e and f) The effect of read-type on expression measured by correlation (e) and gene count (f). Four PTZ-treated samples were chosen that had high, middle, and low gene counts. Single-end fastq libraries were simulated for each sample by randomly sampling the original paired-end dataset (o). Six random simulations were performed for each group, three single-end x 100bp (s100) and three single-end x 50bp (s50). Pearson correlation coefficients between the RSEM aligned log2(TPM) values are plotted in e and the gene counts as a function of TPM threshold are plotted in f. g) Expression of IEGs, *Arc*, *Fos*, and *Egr1* in saline- and PTZ-treated whole-cells. The samples represented by solid dots were analyzed for differences in the activated transcriptome between saline- and PTZ-treated cells.

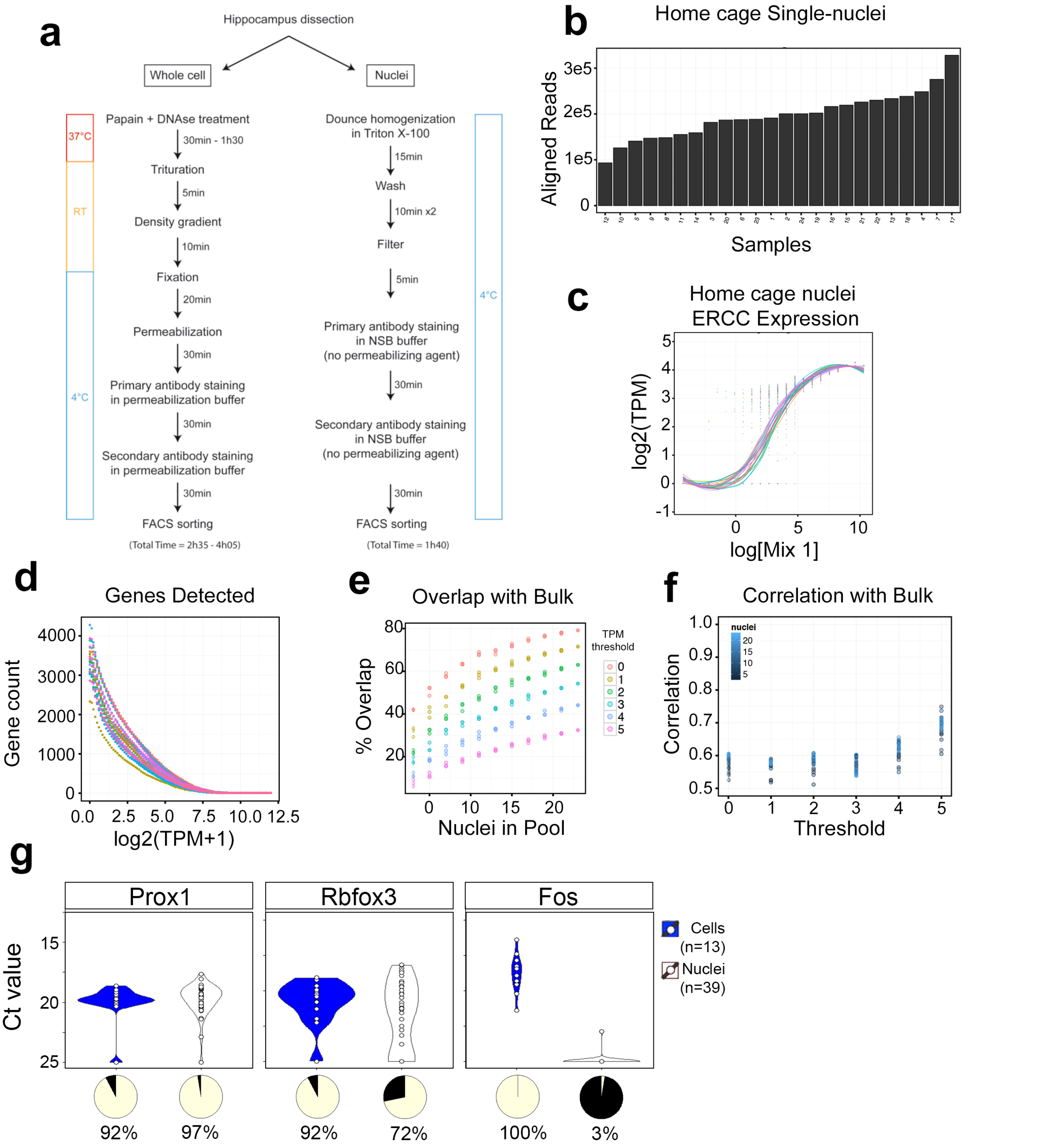

Supplementary Figure 2. Home cage single-nuclei RNA-seq quality measures

a) Comparison of whole cell and nuclei dissociation protocols following mouse hippocampus dissection.

(Left) To dissociate whole cells, minced hippocampus was incubated in a papain solution (20 units/ml

papain, 0.005% DNAse) at 37°C for 30 min-1.5h with gentle agitation, followed by gentle trituration. Dissociated cells were pelleted and resuspended in ovomucoid inhibitor-albumin medium at room temperature (RT). Centrifugation of a single step discontinuous density gradient at RT separated intact whole cells. The pellet containing the intact cells was resuspended in PBS and subsequently fixed with paraformaldehyde (Nuclear Factor Fixation buffer, Biolegend) for 20 min at 4°C. Cells were then permeabilized in Nuclear Factor Permeabilization Buffer (Biolegend) for 30 min, then intranuclear stained with primary and secondary antibodies performed in Nuclear Factor Permeabilization Buffer prior to FACS sorting. (Right) For single nuclei dissociation, dissected hippocampus was immediately placed into a nuclei isolation medium (sucrose 0.25 M, KCl 25 mM, $MgCl_2$ 5 mM, TrisCl 10 mM, dithiothreitol, 0.1 % Triton). Tissue was Dounce homogenized, allowing for mechanical separation of nuclei from cells. The nucleic acid stain Hoechst 33342 (5 µM, Life Technologies) was included in the media to facilitate nuclei visualization. Samples were washed, resuspended in nuclei storage buffer (NSB buffer: sucrose, $MgCl_2$ 5 mM, and TrisCl 10 mM) and filtered. Primary and secondary antibody stainings were performed in NSB buffer prior to FACS sorting. Solutions and samples were kept at 4°C throughout this protocol. b) Number of aligned reads for each HC single-nuclei sample. c) Correlation between the detection of spiked-in ERCCs and their original concentration in Mix1. d) Number of genes detected in each sample as a function of TPM threshold. Each color represents a separate sample (mean 2,185 +/- 377 genes). e) Percentage overlap of genes detected above a given threshold (color) in single-nuclei compared to all genes in bulk nuclei. f) Correlation of genes detected above the TPM threshold (color) in single-nuclei compared to the same genes in bulk. g) qPCR results from whole-cell (N = 13) and single-nuclei (N = 39) preparations. Pie charts indicate the number of samples with detectable expression.

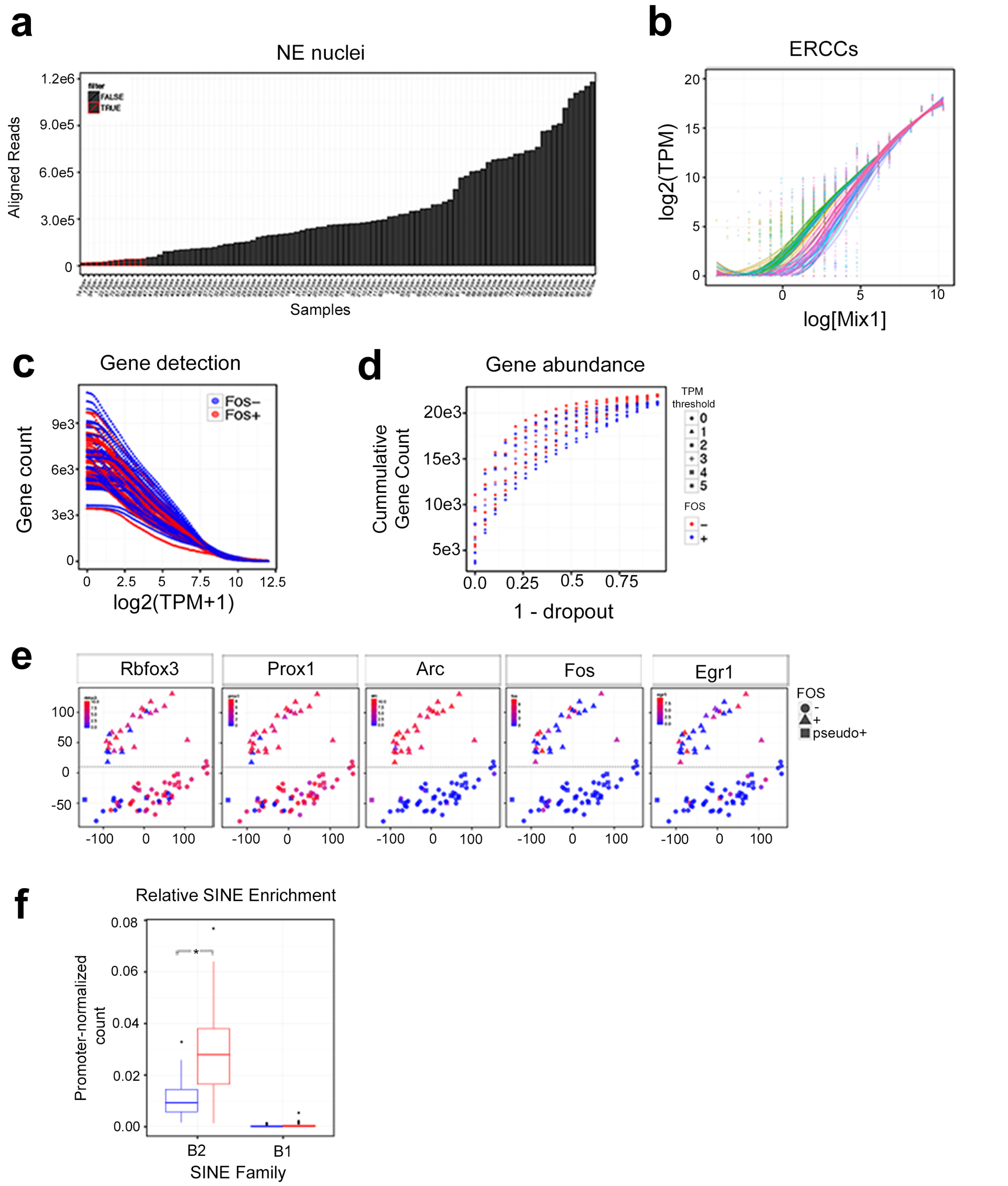

Supplementary Figure 3. Quality of NE nuclei with distinct expression patterns

a) Total aligned reads for each NE nuclei sample; the samples excluded from downstream analysis are colored in red. b) Correlation of the detection of spiked-in ERCC molecules as a function of the

concentration in Mix 1. c) For each sample, the total gene count is plotted for a continuum of TPM thresholds. d) Gene count is plotted versus the number of samples expressing a given gene above the TPM threshold. e) The principal components are plotted as the x- and y-axis with samples colored based on gene expression of *Rbfox3*, *Prox1*, *Arc*, *Fos*, and *Egr1*. f) Reads classified as B2 or B1 SINE retrotransposons which had the start of transcription adjacent to the template-switch oligo were normalized by total reads classified as the given RT element (promoter-normalized count). Counts are plotted for FOS+ (red, $N = 36$) and FOS- nuclei (blue, $N = 43$). Boxplot whiskers extend to 1.5 x the distance between the first and third quartiles of the data (the box), outliers are shown as individual points. * = Student's t-test $p < 5.7e-07$.

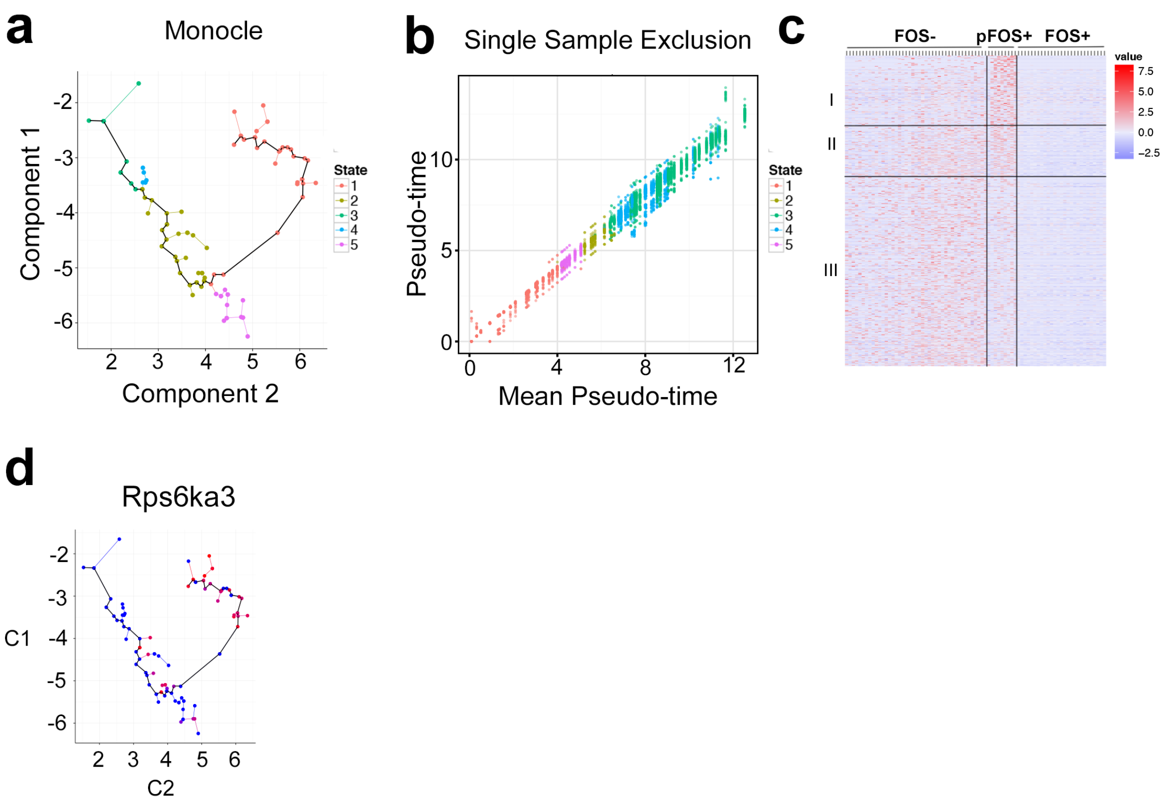

Supplementary Figure 4. Pseudotemporal ordering of NE single-nuclei

a) Independent components plot using the Monocle algorithm. Nuclei are colored by cluster identified using Monocle. b) Each point represents the pseudo-time (color = cluster) calculated for a given sample after single sample exclusion. c) Gene expression specific to pseudoFOS+ (pFOS+) nuclei, I = genes significant only in the comparison between pseudoFOS+ and FOS+, II = genes significant in the comparison between both pseudo-FOS+/FOS+ and FOS-/FOS+, III= genes only significant in the comparison between FOS- and FOS+. d) *Rps6ka3* gene expression within the Monocle trajectory. Red values = high expression, blue values = low expression.

100

GeneName	logFoldChange	GeneName	logFoldChange
Atf3	10.788019	Bloc1s2	-9.570469
Sertad1	10.21171	Calcoco1	-9.288679
Egr4	9.336573	Zfp414	-9.17101
Fos	9.074111	Abhd14b	-9.002621
Myc	8.713386	Tarbp2	-8.921794
Fosb	8.584672	Pcgf1	-8.911985
Nr4a1	8.536112	Irf3	-8.845601
Nr4a2	8.224405	Capn3	-8.806689
Npas4	7.945442	Cc2d1a	-8.714088
Csrnp1	7.863509	Polr2g	-8.558914
Zfp516	7.744754	Zfp239	-8.414245
Jdp2	7.198336	Esrra	-7.679046
Plk3	7.114105	Tfam	-7.482482
Nr4a3	6.752261	Zfp354c	-7.411654
Egr3	6.154286	Nrbf2	-7.36774
Etv3	5.527089	Ice2	-7.336292
Hmga1	5.418522	Brdt	-7.067773
Rbm15	4.464907	Ift74	-6.939597
Sik1	4.28203	Aatf	-6.847781
Rbbp7	3.898561	Vps36	-6.791745
Kdm6b	3.701213	Klf3	-6.693162
Med14	3.555778	Hltf	-6.511412
Pak6	3.331462	Lipe	-6.484055
Tet3	3.317724	Zfp579	-6.465642
Cnot11	3.292378	Prdm5	-6.398878
Skil	2.788771	Nfatc2	-6.368498
Dot1l	2.753235	Otud7b	-6.11169
Fgfr1	2.502289	Fer	-6.041209
Arid5a	2.417917	Birc2	-5.702661
Fbxw7	2.203579	Trim27	-5.586557
Ccnt2	2.149271	Zfp467	-5.583407
Kdm5a	1.633625	Myt1	-5.335186
Uchl5	1.371527	Zbtb5	-5.102086
		Ago1	-4.697533
		Ercc4	-4.664535
		Zfp553	-4.601636
		Nos1	-4.387089
		Csrnp3	-4.287646
		Pbx1	-4.286198

		Ing5	-4.238723
		Zfp2	-4.07929
		Polr1e	-3.953319
		Gcfc2	-3.919166
		C1qbp	-3.869258
		Glmp	-3.752924
		Mdm2	-3.393076
		Pkn1	-3.346777
		Setdb1	-3.277518
		Gabpb2	-3.075843
		Ezh2	-3.022416
		Zfp827	-3.01394
		Bcl9	-3.000494
		Notch1	-2.782151
		Mysm1	-2.727255
		Zbtb49	-2.643727
		Zfp426	-2.573105
		Pygo1	-2.451065
		Myef2	-2.310633
		Lrrfip1	-2.253506
		Stat1	-2.029698
		Tceanc2	-2.019383
		Tox	-1.291768

Supplementary Table 1. Transcription-associated differentially expressed genes

Genes identified as differentially expressed between FOS+ (logFoldChange > 0) and FOS- (logFoldChange < 0) using EdgeR were filtered to retain genes with GO terms related to transcription.
